# GARP2 accelerates retinal degeneration in rod cGMP-gated cation channel β-subunit knockout mice

**DOI:** 10.1038/srep42545

**Published:** 2017-02-15

**Authors:** Marci L. DeRamus, Delores A. Stacks, Youwen Zhang, Carrie E. Huisingh, Gerald McGwin, Steven J. Pittler

**Affiliations:** 1 Departments of Optometry and Vision Science, University of Alabama at Birmingham, 1670 University Blvd, VH 375, Birmingham, AL 35294-0019, USA; 2 Department of Ophthalmology, University of Alabama at Birmingham, 700 18th Street South, Suite 609, Birmingham, AL 35294, USA; 3 Department of Epidemiology, University of Alabama at Birmingham, Ryals Public Health Building, 1665 University Boulevard, Birmingham, AL 35294, USA.

## Abstract

The *Cngb1* locus-encoded β-subunit of rod cGMP-gated cation channel and associated glutamic acid rich proteins (GARPs) are required for phototransduction, disk morphogenesis, and rod structural integrity. To probe individual protein structure/function of the GARPs, we have characterized several transgenic mouse lines selectively restoring GARPs on a *Cngb1* knockout (X1^−/−^) mouse background. Optical coherence tomography (OCT), light and transmission electron microscopy (TEM), and electroretinography (ERG) were used to analyze 6 genotypes including WT at three and ten weeks postnatal. Comparison of aligned histology/OCT images demonstrated that GARP2 accelerates the rate of degeneration. ERG results are consistent with the structural analyses showing the greatest attenuation of function when GARP2 is present. Even 100-fold or more overexpression of GARP1 could not accelerate degeneration as rapidly as GARP2, and when co-expressed GARP1 attenuated the structural and functional deficits elicited by GARP2. These results indicate that the GARPs are not fully interchangeable and thus, likely have separate and distinct functions in the photoreceptor. We also present a uniform murine OCT layer naming nomenclature system that is consistent with human retina layer designations to standardize murine OCT, which will facilitate data evaluation across different laboratories.

Phototransduction, the process by which light stimuli is captured and converted into an electrical response, is initiated in rod and cone photoreceptors of the retina^1^. Within the rod outer segment disks, a photon of light activates the visual pigment, 11-cis-retinal, causing a change in conformation from 11-cis to all-trans. This leads to activation of rhodopsin and subsequently transducin, an intracellular messenger, which activates cGMP phosphodiesterase 6 (PDE6) via displacement of its inhibitory γ-subunits. Activated PDE6 hydrolyzes cytoplasmic cGMP resulting in closure of cGMP-gated cation channels (CNG)^2^,^3^,^4^. Closure of the CNG channels stops the inward Na^+^ and Ca^2+^ current flow and causes a transient hyperpolarization of the photoreceptors, which can be detected as the negative a-wave via electroretinography (ERG)^5^,^6^.

In the rod photoreceptor, the CNG channel is a heterotetramer composed of three α- and one β-subunits which are arranged around a central pore^7^,^8^,^9^. The β-subunit has a bipartite structure, composed of a carboxy-terminal channel-like region and an amino-terminal glutamic acid rich (GARP) region^10^,^11^,^12^. The *Cngb1* locus encodes the β-subunit of the rod CNG cation channel and two associated soluble glutamic acid rich proteins (GARP1, GARP2). The β-subunit and GARP2 have critical roles in phototransduction, disk morphogenesis, and maintenance of outer segment structural integrity^13^,^14^,^15^. Murine GARP1 is a 550 amino acid protein that is four-fold less abundant than the β-subunit, while GARP2 is a 326 amino acid protein that is five-fold more abundant than the β-subunit. As GARP1 is encoded by the first sixteen and GARP2 by the first eleven coding exons of the *Cngb1* locus, these proteins are identical in sequence for 318 amino acids. GARPs are intrinsically disordered proteins^16^,^17^ permitting potential interaction with multiple proteins, within multiple signaling pathways^18^. GARP2 binds to cGMP phosphodiesterase 6 (PDE6) with high affinity and may silence its basal activity up to 80% through interaction with PDE6^16^,^17^. The β-subunit was reported to act as a gating inhibitor and more recently GARP2 was shown to regulate phototransduction gain and recovery^19^. Evidence suggests that soluble GARP2 interacts with peripherin-2 in the rim region of the photoreceptor disk^20^. In addition to their functional role, the GARP region on the β-subunit forms a physical link between the plasma and disk membranes^15^,^20^,^21^. Connections between the plasma and disk membranes have been reported in amphibian, rodent, and bovine rod outer segments based on freeze-fracture electron microscopy^22^,^23^, and cryo-electron tomography^15^,^24^ analyses.

Knockout of the CNG channel β-subunit, while leaving GARPs intact (X26^−/−^), results in loss of function by one month, nearly undetectable levels of CNG α-subunit by two months, and retina degeneration by four months^13^. Knockout of the β-subunit and GARPs^14^ (X1^−/−^) also results in loss of structure and function, including disruption of disk/plasma membrane interactions^15^ and a > 90% decrease in CNG α-subunit levels^14^. However, the loss of function in X26^−/−^ mice occurs 4–5 months earlier and the levels of the CNG α-subunit are significantly lower than in X1^−/−^ mice. To further dissect individual protein structure/function of the GARPs in rods, we have characterized several new transgenic mouse models, which selectively restore the absent GARP proteins to the X1^−/−^ mouse retina. Additionally, we present a system for murine optical coherence tomography (OCT) layer designation, that conforms with recently published human OCT guidelines^25^ and provides the basis for a standardized noninvasive assessment of murine posterior segment structure.

## Results

### Generation of transgenic lines

**Figure 1:**
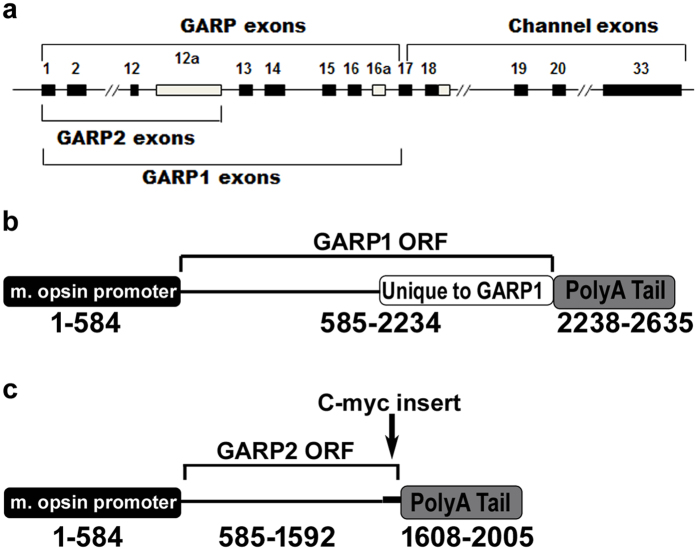
Constructs for transgenic mice expressing GARP1 and GARP2 in the rod photoreceptors. Schematic representation of the CNGB1 locus (**a**), GARP1 (**b**) and GARP2 (**c**) transgene constructs. Expression for both constructs is driven by a 4.8 kb mouse opsin gene promoter^49^ with a c-myc tag added near the C-terminus of GARP2 and a mouse protamine polyA tail^53^.

To analyze the effects of individual gene products of the *Cngb1* locus we generated transgenic lines expressing GARP1 (exons 1–16a, Fig. 1a) and GARP2^19^ (exons 1–12a; Fig. 1b) as previously reported. Expression of GARP1 is under the control of a rod photoreceptor specific 4.4 kb opsin gene promoter^26^. Additionally, for proper expression of the transgene mRNA, a mouse protamine poly-A construct was generated and the PDE6 γ-subunit insert^27^ was replaced with an insert for the full-length murine GARP1 (Fig. 1c). For a summary of the proteins expressed in the different genotypes, see Supplementary Table 1. Characterization of the GARP1 transgenic line was conducted and information relevant to this study is included in Supplementary data (Supplemental Fig. S1).

### A uniform standard for murine OCT layer nomenclature designation

**Figure 2:**
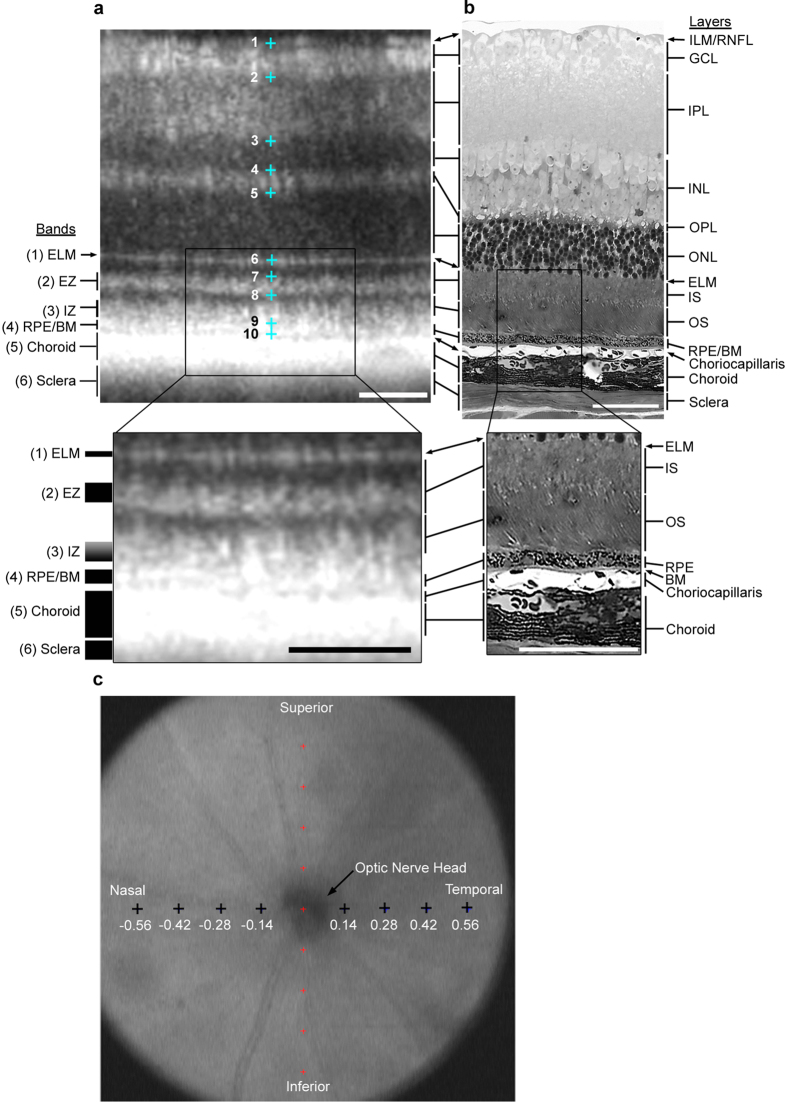
Comparison of (**a**) OCT and (**b**) light histology in a WT mouse, confirming the layer designation in murine OCT images collected using Bioptigen Envisu equipped with InVivoVue^TM^ software. Bands that conform to designations for clinical OCT^25^ are labeled to the left of Panel a. Histological layers are labeled to the right of Panel b. Alignment of histological to OCT layers is indicated by arrows and brackets between Panels a and b. (**a**) Ten horizontally placed marks () were manually placed on the retina, corresponding to specific locations in the retina, using Bioptigen Diver 2.0^TM^ software. This marking scheme was repeated at (**c**) eight equidistant eccentricities (), in one plane with four on either side of the optic nerve. Eccentricity measurements in mm. Scale bars = 50 μm. ILM = inner limiting membrane, RNFL = retinal nerve fiber layer, GCL = ganglion cell layer, IPL = inner plexiform layer, INL = inner nuclear layer, OPL = outer plexiform layer, ONL = outer nuclear layer, ELM = external limiting membrane, IS = inner segment, OS = outer segments, RPE = retinal pigment epithelium, BM = basement membrane, EZ = ellipsoid zone, IZ = interdigitation zone.

While attempts have been made to correlate murine histology and OCT^28^,^29^,^30^,^31^,^32^,^33^,^34^,^35^,^36^,^37^,^38^,^39^,^40^,^41^,^42^,^43^ the labeling nomenclature is often incomplete, does not carefully distinguish OCT bands and histologic structural layers, is inconsistent between groups, or is in conflict with the recently established human OCT nomenclature^25^. Therefore, based on the recommendations of the International Nomenclature for Optical Coherence Tomography panel for human OCT^25^, and our careful alignment of imaging modalities (Fig. 2a and b), we report a uniform standard for rodent OCT layer nomenclature, which we have adopted for this report. This new standard represents the most accurate alignment and layer designation for the mouse to date. Adoption of this nomenclature will create a single standard for OCT layer designation and facilitate comparisons of OCT results from different labs. To create the standard, OCT images of the retinal layers were collected from a four month old C57Bl/6J WT mouse and the eyes were processed for correlative light microscopy. An area of the OCT image (Fig. 2a) was aligned with the light histology image from the same region (Fig. 2b), allowing more accurate identification of the positioning of the layers in the retina. The histology and OCT images were taken from a region approximately 500 μm away from the optic nerve in the nasotemporal region (Fig. 2c). Below the OCT image is an increased magnification view of the outer retina to better discern the band designations and correlation to the corresponding histology image to the right.

In the OCT image (Fig. 2a) the topmost hyperreflective region, between markers 1 and 2, corresponds to the inner limiting membrane/retinal nerve fiber layer and the ganglion cell layer. The adjacent less hyper-reflective region, between markers 2 and 3, corresponds to the inner plexiform layer. The hyporeflective region, between markers 3 and 4, corresponds to the inner nuclear layer. The hyper-reflective region, between markers 4 and 5, corresponds to the outer plexiform layer. The hyporeflective region, between markers 5 and 6, corresponds to the outer nuclear layer. Band (1), indicated by marker 6, corresponds to the external limiting membrane. Band (2), between marker 7 and 8, corresponds to the ellipsoid zone and the inner segment. Band (3), between marker 8 and 9, corresponds to the interdigitation zone which contains the outer segments and microvillus processes of the retinal pigment epithelium. Band (4), indicated by marker 9, corresponds to the retinal pigment epithelium. Band (5), the hyperreflective band directly below marker 10, corresponds to the choroid. Finally, band (6), the less hyperreflective region adjacent to the choroid, corresponds to the sclera.

### GARP2 accelerates retinal degeneration

**Figure 3:**
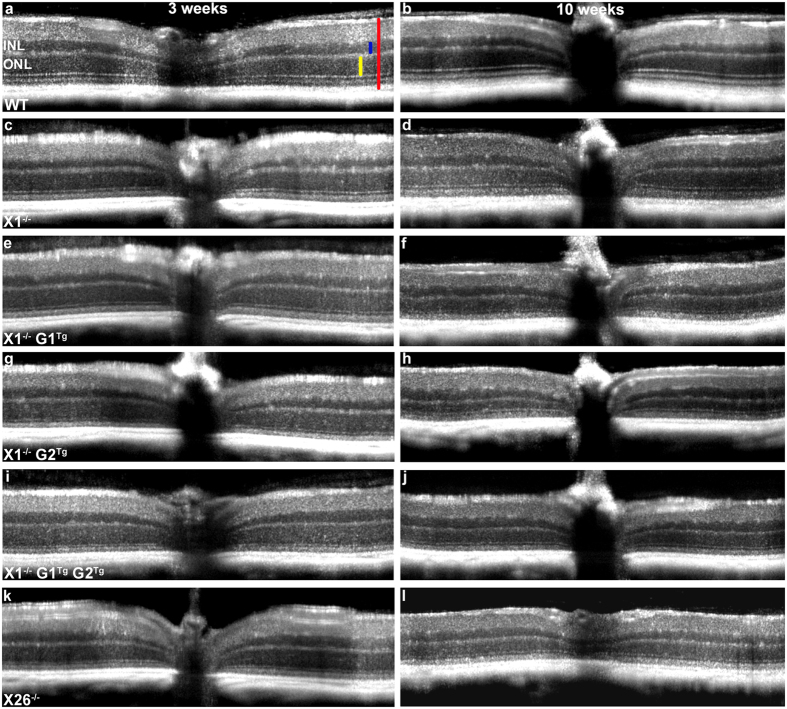
Representative OCT images at 3 (**a,c,e,g,i,k**) and 10 weeks (**b,d,f,h,j,l**) for WT (**a,b**), X1^−/−^ (**c,d**), X1^−/−^ G1^Tg^ (**e,f**), X1^−/−^ G2^Tg^ (**g,h**), X1^−/−^ G1^Tg^ G2^Tg^ (**i,j**), and X26^−/−^ (**k,l**). The images were used to measure outer nuclear layer (ONL) thickness and full retinal thickness (FRT) between genotypes at 3 weeks. Vertical bars indicate full retinal thickness (red), outer nuclear layer (yellow), and inner nuclear layer (blue).

**Figure 4:**
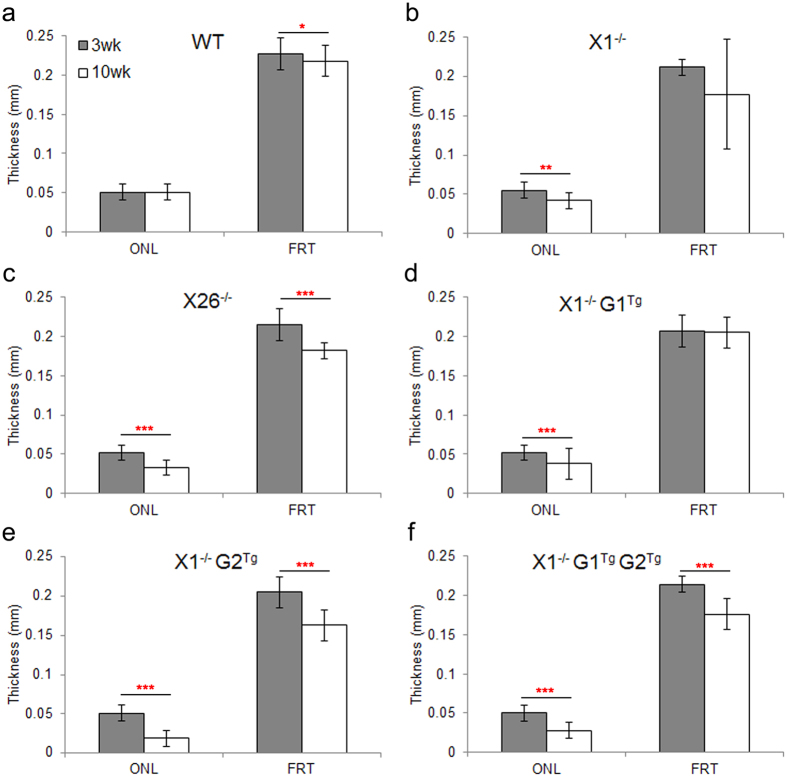
Within group comparison of retinal layer thicknesses over time. Plots of the average OCT measurements of all eccentricities for the outer nuclear layer (ONL) and full retinal thickness (FRT) at 3 and 10 weeks in (**a**) WT, (**b**) X1^−/−^, (**c**) X26^−/−^, (**d**) X1^−/−^ G1^Tg^, (**e**) X1^−/−^ G2^Tg^, and (**f**) X1^−/−^ G1^Tg^ G2^Tg^ mice. There was no difference in WT ONL over time, but WT FRT did decrease slightly. ONL decreased over time, while FRT remained the same in X1^−/−^ and X1^−/−^ G1^Tg^ mice. Both ONL and FRT were reduced in X26^−/−^, X1^−/−^ G2^Tg^, and X1^−/−^ G1^Tg^ G2^Tg^ mice. *p < 0.05, **p < 0.01, ***p < 0.001.

To assess the effects of the transgene alleles on retinal degeneration in X1^−/−^ mice, OCT, light and TEM microscopy were performed. First, the rate of thinning over time was compared within groups, using OCT measurements. As expected, WT showed no change in ONL thickness between three and ten weeks (Figs 3 and 4) in comparison of OCT images. WT did show a slight reduction in FRT between three (0.227 mm ± 0.02) and ten weeks (0.218 mm ± 0.02), which may be due to the initial overextension of rods prior to activation of phagocytosis around the three week period. X1^−/−^ showed reduction in ONL thickness between three (0.055 mm ± 0.01) and ten weeks (0.042 mm ± 0.01), but no change in FRT. Consistent with progressive retinal degeneration in X26^−/−^ mice, OCT analysis of transgenic mice showed significant reduction in ONL thickness (from 0.052 mm ± 0.01 to 0.033 mm ± 0.01) and FRT (from 0.215 mm ± 0.02 to 0.182 mm ± 0.01) over time. The effects of overexpression of G1 on the X1^−/−^ background were also assessed. X1^−/−^ G1^Tg^ showed reduction in ONL thickness between three (0.052 mm ± 0.01) and ten weeks (0.038 mm ± 0.02), but no change in FRT. X1^−/−^ G2^Tg^ showed a reduction in ONL thickness between three (0.051 mm ± 0.01) and ten weeks (0.019 mm ± 0.01), as well as a reduction in FRT (from 0.205 mm ± 0.02 to 0.163 mm ± 0.02). X1^−/−^ G1^Tg^ G2^Tg^ also showed a reduction in ONL thickness between three (0.050 mm ± 0.01) and ten weeks (0.028 mm ± 0.01), as well as a reduction in FRT (from 0.214 mm ± 0.01 to 0.176 mm ± 0.02). All of the genotypes except WT showed a reduction in ONL thickness over time. However, neither X1^−/−^ or X1^−/−^G1^Tg^ showed a reduction in FRT, as was observed for all other genotypes.

**Figure 5:**
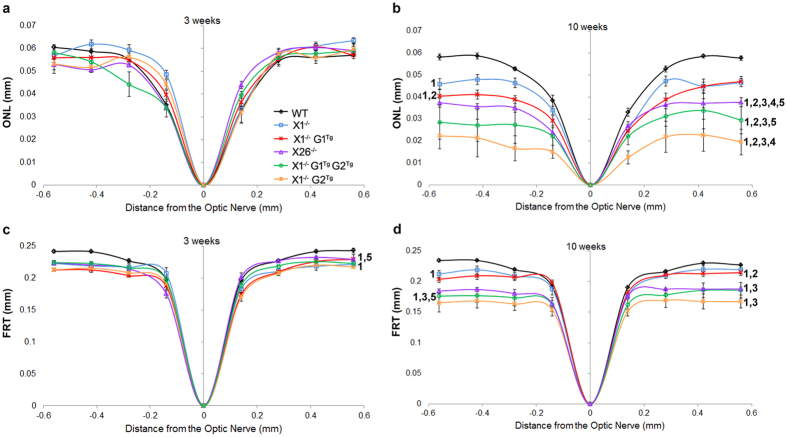
Between groups comparisons of retinal layer thicknesses at each time point. Plots of the average OCT measurements (for 8 eccentricities) of the outer nuclear layer (ONL) at (**a**) 3 weeks and (**b**) 10 weeks and for full retinal thickness (FRT) at (**c**) 3 weeks and (**d**) 10 weeks. At 3 weeks there were no differences in (**a**) ONL thickness, but there was a significant reduction in (**b**) FRT in X1^−/−^, X26^−/−^ X1^−/−^ G1^Tg^, X1^−/−^ G2^Tg^, and X1^−/−^ G1^Tg^ G2^Tg^ mice. At 10 weeks, there was a significant reduction in (**c**) ONL and (**d**) FRT of X1^−/−^ and X26^−/−^ compared to WT. X26^−/−^ showed reduced ONL compared to X1^−/−^ and X1^−/−^ G1^Tg^. There was also a reduction in X1^−/−^ G1^Tg^ ONL compared to WT and X1^−/−^, and a reduction in FRT compared to WT. There was a reduction in ONL of X1^−/−^ G1^Tg^ G2^Tg^ compared to WT, X1^−/−^, X26^−/−^ and X1^−/−^ G1^Tg^, as well as a reduced FRT compared to WT and X1^−/−^ G1^Tg^. Finally, there was a reduction in ONL of X1^−/−^ G2^Tg^ compared to WT, X1^−/−^, X26^−/−^, X1^−/−^ G1^Tg^, and X1^−/−^ G1^Tg^ G2^Tg^, as well as a reduced FRT compared to WT and X1^−/−^ G1^Tg^. Genotypes with a significant reduction compared to WT are indicated by a 1 to the right of the plot. Those with a significant reduction compared to X1^−/−^ are indicated by a 2, to X1^−/−^ G1^Tg^ are indicated by a 3, to X1^−/−^ G1^Tg^ G2^Tg^ are indicated by a 4 and to X1^−/−^ G2^Tg^ are indicated by a 5.

OCT also revealed between group differences in ONL thickness and FRT at each time point (Fig. 5). At three weeks there were no differences in ONL thickness. However, X1^−/−^ (0.212 mm ± 0.01), X1^−/−^ G1^Tg^ (0.207 mm ± 0.02), X26^−/−^ (0.215 mm ± 0.02), X1^−/−^ G2^Tg^ (0.205 mm ± 0.02), and X1^−/−^ G1^Tg^ G2^Tg^ (0.214 mm ± 0.01) all showed reduced FRT compared to WT (0.227 mm ± 0.02). At ten weeks X1^−/−^ (0.042 mm ± 0.01, 0.177 mm ± 0.07) and X26^−/−^ (0.033 mm ± 0.01, 0.182 mm ± 0.01) retinas showed reduced ONL and FRT thickness compared to WT (0.051 mm ± 0.01, 0.218 mm ± 0.02). X26^−/−^ (0.033 mm ± 0.01) retinas showed reduced ONL thickness compared to X1^−/−^ (0.042 mm ± 0.01) and X1^−/−^ G1^Tg^ (0.038 mm ± 0.02). X1^−/−^ G1^Tg^ (0.038 mm ± 0.02) showed reduced ONL thickness compared to WT (0.051 mm ± 0.01) and X1^−/−^ (0.042 mm ± 0.01), as well as reduced FRT (0.205 mm ± 0.02) compared to WT (0.218 mm ± 0.02). X1^−/−^ G2^Tg^ (0.019 mm ± 0.01) showed reduced ONL thickness compared to WT (0.051 mm ± 0.01), X1^−/−^ (0.042 mm ± 0.01), X26^−/−^ (0.033 mm ± 0.01), X1^−/−^ G1^Tg^ (0.038 mm ± 0.02) and X1^−/−^ G1^Tg^ G2^Tg^ (0.028 mm ± 0.01), as well as reduced FRT (0.163 mm ± 0.02) compared to WT (0.218 mm ± 0.02), X26^−/−^ (0.182 mm ± 0.01) and X1^−/−^ G1^Tg^ (0.205 mm ± 0.02). X1^−/−^ G1^Tg^ G2^Tg^ (0.028 mm ± 0.01) showed reduced ONL thickness compared to WT (0.051 mm ± 0.01), X1^−/−^ (0.042 mm ± 0.01), X26^−/−^ (0.033 mm ± 0.01), and X1^−/−^ G1^Tg^ (0.038 mm ± 0.02), as well as reduced FRT (0.176 mm ± 0.02) compared to WT (0.218 mm ± 0.02) and X1^−/−^ G1^Tg^ (0.205 mm ± 0.02). Genotypes expressing GARP2 in the absence of β-subunit (X1^−/−^ G2^Tg^, X1^−/−^G1^Tg^ G2^Tg^, and X26^−/−^) had significant reduction in ONL thickness compared to those that did not express GARP2 (X1^−/−^ and X1^−/−^ G1^Tg^) or expressed GARP2 and β-subunit (WT).

**Figure 6:**
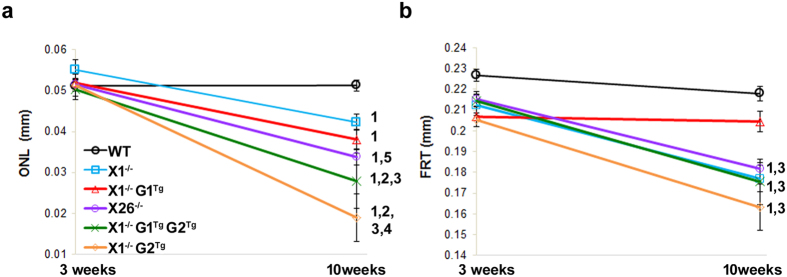
Between groups comparisons of retinal layer thickness over time. Differences in the rate of (**a**) ONL and (**b**) FRT thinning over time. A interaction term was added to the analysis to determine if the changes over time differed by transgenic group. Genotypes with a significant interaction with WT are indicated by a 1 to the right of the plot. Those with a significant interaction with X1^−/−^ are indicated by a 2, with X1^−/−^ G1^Tg^ are indicated by a 3, with X1^−/−^ G1^Tg^ G2^Tg^ are indicated by a 4 and with X1^−/−^ G2^Tg^ are indicated by a 5. The rate of ONL thinning, from highest to lowest, is X1^−/−^ G2 > X1^−/−^ G1^Tg^ G2^Tg^ > X26^−/−^ > X1^−/−^ G1^Tg^ > X1^−/−^ > WT. The rate of FRT thinning, from highest to lowest, is X1^−/−^ G2^Tg^, X1^−/−^ G1^Tg^ G2^Tg^, X1^−/−^, and X26^−/−^** > **X1^−/−^ G1^Tg^ and WT.

The rate of thinning over time was compared between groups, a measurement of the interaction between time and genotype (Fig. 6). In general, when a plot of change in thickness over time for one genotype is parallel to the plot of another genotype, there is likely not a significant interaction (difference in rate of thickness change) between change in thickness over time by genotype. However, if the two plots are not parallel, there is likely a significant interaction (difference in rate of thickness change). The steeper the downward slope of the line, the greater the rate of thinning, indicating more rapid thinning or cell loss of the layer. Thus, the rate of ONL thickness reduction (Fig. 6a) is greater over time in X1^−/−^, X26^−/−^ and X1^−/−^ G1^Tg^ than in WT (p < 0.001). ONL thinned more rapidly in X1^−/−^ G2^Tg^ than in WT, X1^−/−^, X26^−/−^, X1^−/−^ G1^Tg^, or X1^−/−^ G1^Tg^ G2^Tg^. ONL thinned more rapidly in X1^−/−^ G1^Tg^ G2^Tg^ than in WT, X1^−/−^, or X1^−/−^ G1^Tg^. Therefore, the greatest rate of ONL thinning is in X1^−/−^ G2^Tg^. There is ONL thinning in X1^−/−^ G1^Tg^, but at a much slower rate than in X1^−/−^ G2^Tg^. Expression of both GARP1 and GARP2 in X1^−/−^ G1^Tg^ G2^Tg^ shows a slower rate of ONL thinning than X1^−/−^ G2^Tg^, but faster than X1^−/−^ G1^Tg^. Thus, in order of decreasing rates of thinning, the comparative rate of thinning per genotype is X1^−/−^ G2 > X1^−/−^ G1^Tg^ G2^Tg^ > X26^−/−^ > X1^−/−^ G1^Tg^ > X1^−/−^ > WT. The only difference between X26^−/−^ and X1^−/−^ G1^Tg^ G2^Tg^, is that the latter expresses more GARP1 than the former. This indicates that co-expression of GARP2 along with increased GARP1 (X1^−/−^ G1^Tg^ G2^Tg^) results in increased rates of thinning. However, increased expression of GARP1 without GARP2 (X1^−/−^ G1^Tg^) has a lower rate of thinning than endogenous expression of GARP1 with GARP2 (X26^−/−^), suggesting that GARP2 expression has the strongest effect on the rate of thinning.

The rate of FRT thinning (Fig. 6b) is greater in X1^−/−^ and X26^−/−^ than in WT or X1^−/−^ G1^Tg^. The rate of FRT thinning is greater in X1^−/−^ G2^Tg^ than WT or X1^−/−^ G1^Tg^. Finally, the rate of FRT thinning is greater in X1^−/−^ G1^Tg^ G2^Tg^ than WT or X1^−/−^ G1^Tg^. Considering all genotypes, the rate of thinning from fastest to slowest is X1^−/−^ G2^Tg^, X1^−/−^ G1^Tg^ G2^Tg^, X1^−/−^, and X26^−/−^ > X1^−/−^ G1^Tg^ and WT. The only genotype that did not have faster FRT thinning than WT was X1^−/−^ G1^Tg^.

**Figure 7:**
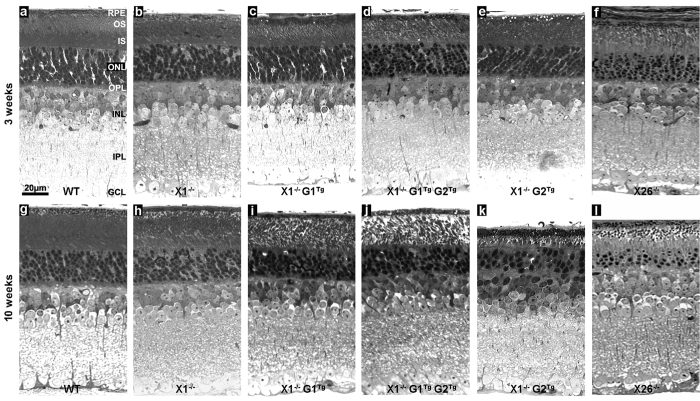
Representative light microscopy images of WT (**a,g**), X1^−/−^ (**b,h**), X1^−/−^ G1^Tg^ (**c,i**), X1^−/−^ G1^Tg^ G2^Tg^ (**d,j**), X1^−/−^ G2^Tg^ (**e,k**), and X26^−/−^ (**f,l**) at 3 (**a–f**) and 10 weeks (**g–l**) pn. At 10 weeks pn WT has 10 rows of nuclei in the outer nuclear layer (ONL) compared to 8–9 rows in all other genotypes. At 10 weeks, WT ONL has 9–10 rows of nuclei while X1^−/−^ and X1^−/−^ G1^Tg^ have 6–7 rows, X1^−/−^ G1^Tg^ G2^Tg^ has 5–6 rows, X26^−/−^ has 4 rows and X1^−/−^ G2^Tg^ only has 3 rows. In addition, photoreceptor outer segments are more disorganized and show misalignment in X1^−/−^; X1^−/−^ G1^Tg^, and X1^−/−^ G1^Tg^ G2^Tg^, while they are almost completely gone in X1^−/−^ G2^Tg^. RPE = retinal pigment epithelium, OS = outer segment, IS = inner segment, OPL = outer plexiform layer, INL = inner nuclear layer, IPL = inner plexiform layer, GCL = ganglion cell layer, scale bar = 20 μm.

To validate OCT results, histologic analysis was performed on each genotype at three and ten weeks (Fig. 7). At three weeks, WT ONL is 0.033 mm ± 0.002, compared to 0.032 mm ± 0.002 for X1^−/−^, 0.039 mm ± 0.005 for X26^−/−^, 0.040 mm ± 0.003 for X1^−/−^ G1^Tg^, 0.042 mm ± 0.003 for X1^−/−^ G2^Tg^ and 0.037 mm ± 0.003 for X1^−/−^ G1^Tg^ G2^Tg^. At ten weeks WT ONL is 0.036 mm ± 0.002, compared to 0.032 mm ± 0.003 for X1^−/−^, 0.026 mm ± 0.003 for X26^−/−^, 0.029 ± 0.004 for X1^−/−^ G1^Tg^, 0.021 mm ± 0.002 for X1^−/−^ G1^Tg^ G2^Tg^, and 0.025 mm ± 0.004 for X1^−/−^ G2. Similarly to OCT results, there was a significant within group difference between 3 and 10 weeks for all genotypes except WT and X1^−/−^. While OCT results did show a significant thinning for X1^−/−^ between 3 and 10 weeks, this was the smallest difference and the least statistically significant change of all of the genotypes. Additionally, there were between group differences for ONL thickness at 10 weeks. OCT results showed a significant thinning in ONL at 10 weeks in X26^−/−^, X1^−/−^ G1^Tg^ G2^Tg^, and X1^−/−^ G2^Tg^ retinas compared to WT. However, histology measurements showed a significant thinning in ONL at 10 weeks for all genotypes compared to WT. At 3 weeks, WT FRT is 0.182 mm ± 0.006, compared to 0.179 mm ± 0.005 for X1^−/−^, 0.245 mm ± 0.025 for X26^−/−^, 0.233 mm ± 0.013 for X1^−/−^ G1^Tg^, 0.220 mm ± 0.012 for X1^−/−^ G2^Tg^, and 0.200 mm ± 0.012 for X1^−/−^ G1^Tg^ G2^Tg^. At 10 weeks, WT FRT is 0.205 mm ± 0.010, compared to 0.197 mm ± 0.014 for X1^−/−^, 0.190 mm ± 0.013 for X26^−/−^, 0.179 mm ± 0.011 for X1^−/−^ G1^Tg^, 0.201 mm ± 0.020 for X1^−/−^ G2^Tg^, and 0.155 mm ± 0.006 for X1^−/−^ G1^Tg^ G2^Tg^. While OCT results only showed a significant FRT thinning over time in WT, X26^−/−^, X1^−/−^ G2^Tg^, and X1^−/−^ G1^Tg^ G2^Tg^, histology measurements showed significant FRT thinning over time in all genotypes. OCT results at 10 weeks showed a significantly thinner FRT for X26^−/−^, X1^−/−^ G1^Tg^, X1^−/−^ G1^Tg^ G2^Tg^, and X1^−/−^ G2^Tg^ compared to WT. Similar results were observed for histology measurements, except histology did not show a difference in FRT between WT and X26^−/−^ retinas. Overall, histology measurements of ONL and FRT confirmed OCT observations. In addition, histology also showed that photoreceptor outer segments are less uniform and more disorganized in X1^−/−^, X1^−/−^ G1^Tg^ and X1^−/−^ G1^Tg^ G2^Tg^. Photoreceptor outer segments are almost completely lost in X1^−/−^ G2^Tg^ at 10 weeks. At 10 weeks, GARP2 accelerates thinning; however GARP1 does not affect the rate of thinning and partially counteracts the effect of GARP2 in the X1^−/−^ G1^Tg^ G2^Tg^ hybrid.

High magnification electron microscopy (EM) images revealed structural changes as early as three weeks in X1^−/−^, X1^−/−^ G1^Tg^, X1^−/−^ G2^Tg^, and X1^−/−^ G1^Tg^ G2^Tg^ retinas (Supplemental Fig. S2). In WT, OS were tightly packed, in a uniformly parallel orientation. In the X1^−/−^ and transgenic mice rod outer segments were less tightly packed than in WT retina. In X1^−/−^ G1^Tg^ G2^Tg^ and X1^−/−^ G2^Tg^ retinas the photoreceptors were severely shortened and photoreceptor atrophy is apparent. By ten weeks, OS are widely spaced, disordered, and truncated in X1^−/−^ G1^Tg^, X1^−/−^ G1^Tg^ G2^Tg^, and X1^−/−^ G2^Tg^ mice. In X1^−/−^ G2^Tg^ mice photoreceptor atrophy is apparent, with very little photoreceptor remaining. Due to progressive degeneration for all mutant genotypes, clear IS/OS junctions are not discernible.

**Figure 8:**
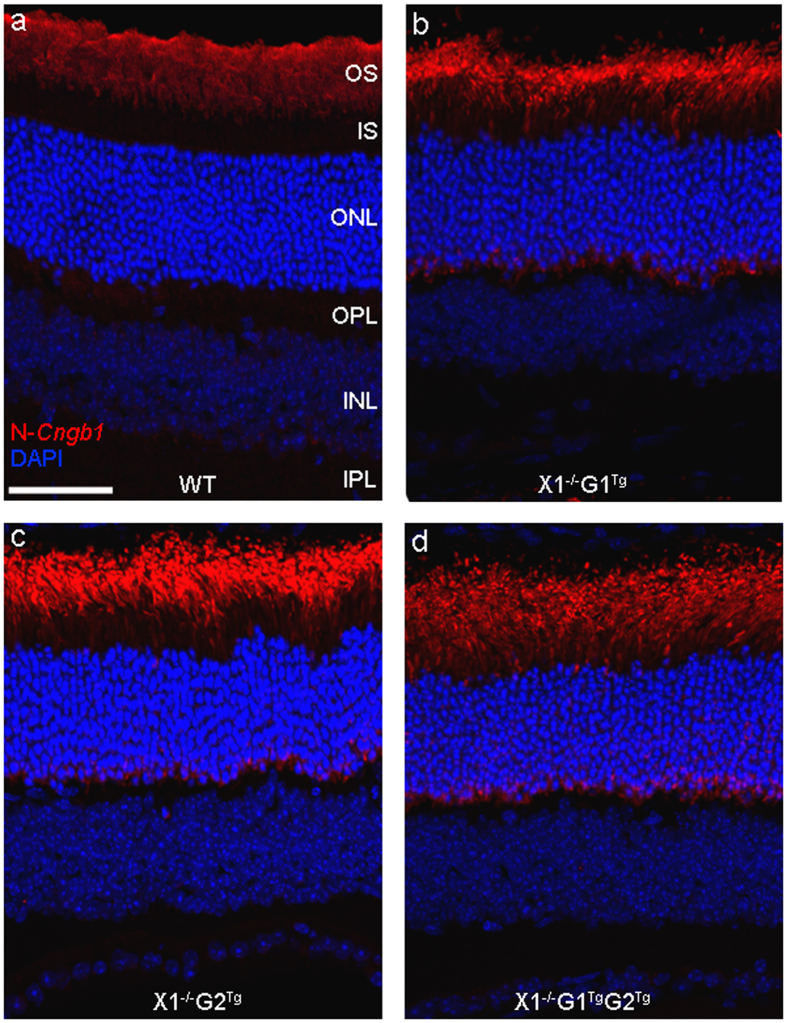
Representative expression of *Cngb1* proteins in 4–6 week old (**a**) WT, (**b**) X1^−/−^ G1^Tg^, (**c**) X1^−/−^ G2^Tg^, and (**d**) X1^−/−^ G1^Tg^ G2^Tg^ retinas. GARP expression was primarily observed in OS, with minimal expression in IS in the transgenic retinas. Scale bar = 50 μm.

Because there is an ongoing degeneration it could not be assumed that the transgene expressed GARPs are properly localizing in photoreceptors. Therefore, we determined the localization of GARPs in WT and the transgenic animals. Using an antibody that recognizes the N-terminus of the β-subunit and GARPs (N-*Cngb1*) at 1 month postnatal (Fig. 8), showed expression of GARPs in the OS in all genotypes. Additionally, in the X1^−/−^ G1^Tg^, X1^−/−^ G2^Tg^, and X1^−/−^ G1^Tg^ G2^Tg^ animals, minimal expression in the IS was observed. Negative controls (not shown), without the N-*Cngb1* primary antibody showed no signal in any genotype.

### Quantitative analysis of GARP levels

**Figure 9:**
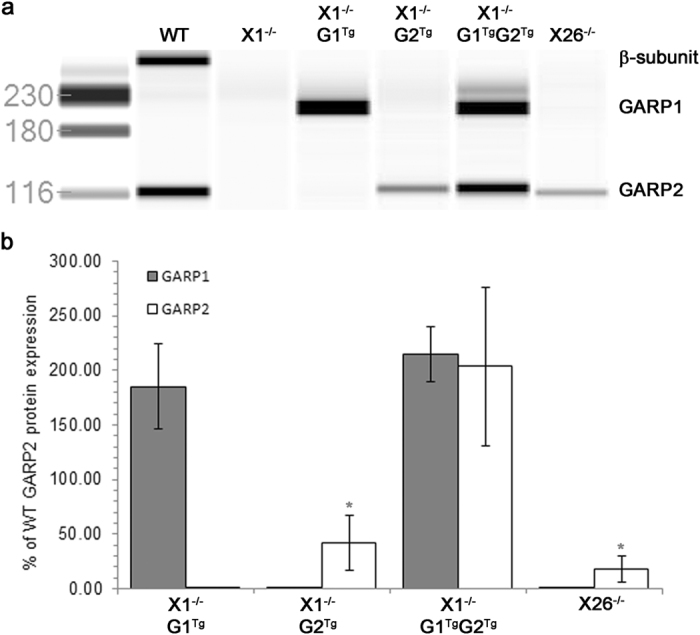
Quantitation of GARPs in retinal homogenates. (**a**) Representative digital image showing antibody binding using an antibody specific for the N-terminus of the β-subunit (~280 kDa), GARP1 (~220 kDa), and GARP2 (~120 kDa). (**b**) Graph representing expression levels of GARP1 and GARP2 as % of WT GARP2 protein expression in X1^−/−^G1^Tg^, X1^−/−^G2^Tg^, X1^−/−^G1^Tg^G2^Tg^, and X26^−/−^ mice. GARP2 expression was significantly lower in X1^−/−^G2^Tg^ and X26^−/−^ than in X1^−/−^G1^Tg^G2^Tg^ mice (*p < 0.05).

To compare proteins levels of channel β-subunit, GARP1, and GARP2 in the various genotypes, automated capillary-based Western analysis was performed, using an internal standard for normalization. Similar to previously published results^14^ using the N-*Cngb1* antibody, the channel β-subunit and GARP2 are readily detectable, but GARP1 was not detectable in WT retina (Fig. 9, lane WT). In contrast, in X1^−/−^ retina homogenates, no channel or GARP2 protein is detectable, consistent with the X1^−/−^ being a true null (lane X1^−/−^). When GARP1 is overexpressed without GARP2 (X1^−/−^G1^Tg^) or co-expressed with GARP2 (X1^−/−^G1^Tg^G2^Tg^) it was readily apparent. GARP2 was easily detected in WT, X1^−/−^G2^Tg^, X1^−/−^G1^Tg^G2^Tg^, and X26^−/−^ mice. Protein expression is shown as the percent of GARP2 expression in WT retina (Fig. 9b). While GARP1 was not detectable in WT, X1^−/−^, or X26^−/−^ mice, GARP1 was expressed at 185% (±39%) in X1^−/−^G1^Tg^ mice and 215% (±26%) in X1^−/−^G1^Tg^G2^Tg^ mice. GARP2 was expressed at 42% (±25%) in X1^−/−^G2^Tg^ mice, 204% (±72%) in X1^−/−^G1^Tg^G2^Tg^ mice, and 18% (±12%) in X26^−/−^ mice. Despite a 30% increase in GARP1 expression in X1^−/−^G1^Tg^G2^Tg^ compared to X1^−/−^G1^Tg^ mice, this difference was not statistically significant. However, there was significantly lower expression of GARP2 in X1^−/−^G2^Tg^ and X26^−/−^ mice compared to X1^−/−^G1^Tg^G2^Tg^ mice. Despite a 20% increase in GARP2 expression in X1^−/−^G2^Tg^ mice compared to X26^−/−^ mice, this difference was not statistically significant.

### Structural loss correlates with functional loss

To correlate functional loss with structural loss, scotopic electroretinography was performed at three and ten weeks. At 3 weeks (Supplementary Table 2), the WT mouse a-wave (358 μv ± 32) and b-wave (953 μv ± 55) scotopic responses to a 25 cd*s/m^2^ flash were significantly higher than X1^−/−^ (34 μv ± 3, 377 μv ± 43), X26^−/−^ (21 μv ± 12, 163 μv ± 21), X1^−/−^ G1^Tg^ (27 μv ± 8, 282 μv ± 21), X1^−/−^ G1^Tg^ G2^Tg^ (40 μv ± 15, 254 μv ± 91) and X1^−/−^ G2^Tg^ (26 μv ± 9, 261 μv ± 128). In addition, the X1^−/−^ a-wave was significantly higher than X1^−/−^ G2^Tg^, and the b-wave was significantly higher than X1^−/−^ G2^Tg^ and X1^−/−^ G1^Tg^ G2^Tg^. At 10 weeks (Supplementary Table 3), similar results were observed in that the WT mouse a-wave (358 μv ± 39) and b-wave (878 μv ± 65) were significantly higher than X1^−/−^ (32 μv ± 5, 202 μv ± 46), X26^−/−^ (24 μv ± 2, 141 μv ± 27), X1^−/−^ G1^Tg^ (14 μv ± 3, 133 μv ± 24), X1^−/−^ G1^Tg^ G2^Tg^ (19 μv ± 3, 114 μv ± 12) and X1^−/−^ G2^Tg^ (18 μv ± 2, 105 μv ± 14). These data are consistent with a negative effect of GARP2 on rod function in the absence of the β-subunit. Scotopic responses were also measured at lower intensities (0.0001–10 cd*s/m^2^) and showed similar results as the saturating 25 cd*s/m^2^ flash (see Supplementary Tables 2 and 3).

## Discussion

OCT is a very useful tool to evaluate changes in retinal structure in a mouse model. Recently, a consensus for normal human OCT terminology was agreed upon by the International Nomenclature for Optical Coherence Tomography [IN • OCT] Panel^25^. Similar establishment of consistent labeling nomenclature for mouse OCT would be useful to ensure accurate, consistent and cross-lab comparable measurements and proper identification of regions in mouse studies. Presently, reports of mouse OCT retina layers in the literature vary widely. Some studies report incomplete labeling of OCT scans, both with^28^,^30^,^44^ and without^31^,^32^,^33^,^35^,^36^,^45^ histological comparisons. Other reports document very specific labeling of retina layers with clearly defined regions, again with^29^,^37^,^38^,^39^,^40^,^46^ and without^41^,^42^,^43^ histological comparisons, but the labeling of layers differs. For example, the GCL is reported alone^28^,^29^,^30^,^37^,^39^,^44^,^45^, grouped with the NFL^38^,^40^,^46^, grouped with the IPL^41^,^42^, or grouped with both the NFL and IPL^31^,^32^. The IS and OS are sometimes reported separately^30^,^39^,^44^ and sometimes grouped together^28^,^33^,^38^,^40^,^43^,^46^. Identification of Bruch’s membrane, ELM, and the interdigitation zone is sporadic and inconsistent. ELM is sometimes grouped with the IS/OS^41^,^42^ and sometimes referred to as OLM^39^. Bruch’s membrane (BM) is rarely reported, but when it is, it’s labeled separately from the RPE^29^, conflicting with the consensus reached in human retina which determined that RPE and BM could not be differentiated in OCT^25^.

In the current study we have ensured careful identification of regions within the retina in OCT images by comparing aligned OCT and light histology images in a WT mouse, using the established human OCT nomenclature as a guide^25^. Although carefully aligned, there were slight differences in scale between fixed tissue images and OCT images. These minor discrepancies can likely be attributed to differences in resolution between the two techniques, and fixed versus *in vivo* tissue. Accurate assessment of regions within the OCT allowed for accurate measurements of retinal thickness and outer nuclear layer thickness with minimal variability. We propose that this nomenclature be adopted going forward for proper description of the layers for mouse OCT, and similar nomenclature can be adopted for other species.

Using the mouse OCT nomenclature described in Fig. 2, X1^−/−^ G1^Tg^, X1^−/−^ G2^Tg^, and X1^−/−^ G1^Tg^ G2^Tg^ show ONL thinning in comparison to WT at ten weeks, similar to previous measurements of X1^−/−^ mice, but the rate of thinning is accelerated in mice carrying the GARP2 allele. Co-expression of GARP1 and GARP2 on an X1^−/−^ background slows thinning compared to X1^−/−^ G2^Tg^, but thinning is still faster than observed in X1^−/−^ G1^Tg^ mice indicating the negative effect of GARP2 is dominant over GARP1. In addition, the fact that X1^−/−^ G1^Tg^ at ~100 fold overexpression does not accelerate thinning as fast as any genotype with GARP2, indicates differing roles for these proteins. Given these results, it is also likely that endogenous levels of GARP1 are unlikely to cause degeneration on the X1^−/−^ background as has been observed with GARP2 expression on the X1^−/−^ background (X1^−/−^ G2^Tg^).

As expected, photoreceptor (a-wave) and bipolar cell function (b-wave), measured via ERG, were reduced in all genotypes compared to WT. In addition, photoreceptor and bipolar cell function (attenuated a- and b-waves in ERG) were consistent with OCT findings, with the greatest attenuation in X1^−/−^ G2^Tg^ mice. Co-expression of GARP1 and GARP2 on the X1^−/−^ reduced this attenuation, but it was still greater than the attenuation observed in X1^−/−^ G1^Tg^. These results provide further evidence that the negative effect of GARP2 is dominant over GARP1, and suggests a possible interaction between GARP1 and GARP2. This also further supports the conclusion that GARP2, in the absence of the β-subunit, drives functional and structural decline in the retina. Finally, coupled with the structural data, this also suggests GARP1 and GARP2 have distinct functions in the rods.

In WT mice, GARP1 is 20-fold less abundant than GARP2 and four-fold less than the CNG β-subunit^16^. Due to the normally low endogenous WT levels of GARP1 and the 318 amino acid sequence common to both GARP proteins, it would be reasonable to speculate the proteins would be interchangeable in function. GARP1 may be redundant and not necessary for rod structure/function. In order to address whether GARPs are interchangeable or have separate functions we examined several mouse models altering the expression of these proteins. We used a GARP1 transgene that shows a 100-fold increase in expression in mice on a WT background, compared to GARP1 expression in WT (see Supplementary Fig. 1). This strain was crossed into the X1^−/−^ background which increased the GARP1 levels to approximately endogenous levels of GARP2 expression. A GARP2 transgene (at endogenous levels of protein when expressed on a WT background) was also crossed onto the X1^−/−^ background. In addition, the two GARP expressing animals were then crossed to enable examination of expression of the two transgenes in the absence of the CNG β-subunit. Finally, endogenous GARP1 and GARP2 were both expressed in the X26^−/−^ mouse which only lacks the CNG β-subunit. If the two proteins have identical function one would expect identical phenotypes when expressed in various genetic backgrounds where phenotypes are discernible. Furthermore, one would expect the co-expression of both GARPs to further accelerate the thinning. The ONL in X26^−/−^ mice thins more slowly than in X1^−/−^ G2^Tg^.

Previous studies have examined GARP/β-subunit expression in WT, X1^−/−^, X26^−/−^, and a G2 overexpression mouse^13^,^14^,^19^,^47^. In WT, X26^−/−^, and the G2 overexpression mouse, GARP/β-subunit expression is confined to the OS with variable OPL signal. Unsurprisingly, GARP/β-subunit expression is absent in the X1^−/−^ mouse. GARP expression in the X1^−/−^ G1^Tg^, X1^−/−^ G2^Tg^, and X1^−/−^ G1^Tg^ G2^Tg^ mice was confined primarily to the OS, with minimal expression in the IS and variable signal in OPL. This shows that the transgene, even when overexpressed, primarily localizes to the OS.

Total GARP2 protein expression in X26^−/−^ mice is ~18% of the endogenous GARP2 expression, while GARP2 expression in X1^−/−^ G2^Tg^ mice is ~42%. This higher level of GARP2 expression could explain why the ONL in X26^−/−^ mice thins more slowly than in X1^−/−^ G2^Tg^. Crossing the X1^−/−^ G1^Tg^ (185% of WT GARP2 expression) and X1^−/−^ G2^Tg^ (42% of WT GARP2 expression) mice results in offspring that express GARP2 at ~200% of WT GARP2 levels. X1^−/−^ G1^Tg^ mice do not have significantly faster ONL thinning than X26^−/−^, but the X1^−/−^ G1^Tg^ G2^Tg^ mice (with the 200% increases in GARP2 expression) do have significantly faster rates of thinning than X26^−/−^ mice. However, the increased GARP2 expression in X1^−/−^ G1^Tg^ G2^Tg^ is not enough to increase the rate of thinning compared to X1^−/−^ G2^Tg^ mice, which have lower levels of GARP2. Thus, the presence of GARP1 must be slowing the rate of thinning. This also indicates differing roles for GARP1 and GARP2 and the possibility that increased expression of GARP1 is somehow involved in increasing expression of GARP2. Taken together, our results lead us to conclude that GARP1 and GARP2 are not interchangeable as they do not produce identical phenotypes, nor are they additive in effect, and thus these proteins may have distinct and separate roles in the rod photoreceptor.

One possible mechanism for the acceleration of degeneration in our GARP transgenic mice could be inhibition of α-subunit transport (in the absence of the β-subunit) from IS to OS which is consistent with the significantly lower α-subunit levels found in X26^−/−^13 mice expressing both GARPs compared to X1^−/−^14 mice that do not express GARPs. This is also consistent with the results reported here showing some localization of GARPs in the IS of the transgenic mice. Furthermore, it is has already been established that GARPs can bind to the α-subunit^48^. This may also indicate a role for the β-subunit in channel transport to the outer segment, similar to what was reported for the olfactory β-subunit^14^, a splice variant of the rod β-subunit. The inability of GARP1 to generate as robust a phenotype as GARP2 could be due to a reduced ability to bind the α-subunit and inhibit transport because of its additional β-subunit sequence^48^.

## Methods

### Animal generation and husbandry

An exon 1, 2 and predicted promoter Cngb1 knockout null allele (X1^−/−^)^14^ and an exon 26 knockout retaining GARP expression (X26^−/−^)^13^ were previously described^13^, and are being maintained by crossing homozygous knockout mice. Transgenic mice were generated to express GARP1 (G1^Tg^, 100-fold overexpressed) using a 4.4 kb opsin promoter, mouse protamine poly-A construct generated by replacing the PDE6 γ-subunit insert^49^ with a full-length murine GARP1 insert (Fig. 1c). GARP2 (G2^Tg^, construct shown in Fig. 1b) transgenic mice were previously described^19^. For these studies, GARP2 mice expressing myc-tagged GARP2 at WT endogenous levels (Line 1 instead of overexpressing Line 6) were used^19^. X1^−/−^ G1^Tg^ G2^Tg^ mice carrying both alleles were generated by crossing G1^Tg^ and G2^Tg^ transgenic mice on a homozygous X1^−/−^ background and identifying the presence of both alleles by PCR genotyping (see below). All animal lines were made congenic on a C57BL/6J background. All animal use protocols were approved by the University of Alabama at Birmingham (UAB) Institutional Animal Care and Use Committee and are consistent with the Association for Research in Vision and Ophthalmology guidelines for the use of animals in research. All animals were maintained on a standard 12/12-hour light/dark cycle, fed standard rodent chow, and housed with standard rodent bedding.

### PCR genotyping

Genomic DNA was extracted from mouse tails using proteinase K (10 mg/ml; Sigma Chemical Co., St. Louis, MO) in digestion buffer consisting of 5 mM EDTA, 200 mM NaCl, 100 mM Tris-HCl, and 0.2% SDS. Tails were digested overnight at 55 °C. 100% ethanol was added to the lysate, which was then centrifuged at 16,000 × g for thirty minutes, washed with 70% ethanol, centrifuged at 16,000 × g for twenty minutes, and then the DNA pellet was dissolved in TE buffer, pH 8.0. PCR was performed using the following cycling parameters: 94 °C denaturation, five minutes, for one cycle; thirty five cycles of 94 °C, thirty seconds; for GARP1, 58 °C, thirty seconds; for GARP2, 62 °C, forty five seconds; 72 °C, fifty seconds; and one cycle of 72 °C for seven minutes. Primers sequences for X26^−/−^ 13 and X1^−/−^ 14 have been previously described. Primer sequences for detection of the GARP1 transgene (750 bp) were (Sense) 5′-GGGAGGCCACAAACTCAACA-3′ and (Antisense) 5′-CGCAGGAGTTTTGATGGACT-3′ and for the GARP2 transgene (679 bp) were (Sense): 5′-GCTGGTCCCAGCCTTCA-AGAGA-3 and (Antisense) 5′-CTCTTCTGAGATGAGTTTTTGTTCGGTC-3′.

### Optical coherence tomography (OCT)

Spectral domain OCT (840 nm; Envisu Class-R, Bioptigen, Inc, Morrisville, NC) was performed to visualize the posterior segment in living mice. The Bioptigen OCT instrument has a transverse resolution of 2.5 μm and an axial resolution of 1.6 μm. Animals were anesthetized with intraperitoneal injection of ketamine/xylazine (Bioniche Teoranta; Inverin, Co. Galway, Ireland) at 100 mg/kg and 10 mg/kg body weight, respectively. Pupils were dilated using 1% tropicamide (Bausch & Lomb; Rochester, NY) and 0.5% proparacaine (Falcon Pharmaceuticals; Fort Worth, TX), and artificial tears (Systane Ultra; Alcon OTC; Houston, TX) were applied frequently, with saline wash throughout to maintain corneal clarity. Scans were captured with the beam centered on the optic nerve. Both horizontal and vertical linear scans were obtained with Bioptigen InVivoVue^TM^ 1.4 software (Bioptigen, Inc., Durham, NC, USA. Scan parameters were as follows: (scan parameter one) rectangular volume scan of 1.4 mm diameter with one thousand A-scans/B-scan, one hundred B-scans/volume, and one frame/B-scan or (scan parameter two) rectangular volume scan of 1.6 mm in diameter with one thousand A-scans/B-scan, three B-scans/volume, and forty eight frames/B-scan. The one thousand A-scan frames collected in parameter two were averaged to reduce noise and increase signal. These averaged images were then converted to bmp files. To determine the ONL thickness, the difference in distance between the outer plexiform layer (OPL) and the external limiting membrane (ELM) was calculated. To determine the FRT, the difference in distance between the inner limiting membrane (ILM) and retinal pigment epithelium (RPE) was calculated. Measurements of ONL thickness and FRT were performed with Bioptigen Diver 2.4 software on retinal OCT images, captured using scan parameter one, of three week and ten week old mice at eight equidistant eccentricities (Fig. 2a), in one plane, from the optic nerve head (four eccentricities on each side of the optic nerve). At each eccentricity ten horizontally placed marks were manually placed on the OCT image, to indicate a specific location in the retina (Fig. 2c). ONL thickness was measured as the distance between mark five and six, while FRT was determined as the distance between marks one and ten. Representative averaged bitmap images (Fig. 3) were taken from images captured under scan parameter two described above, brightness/contrast was adjusted in Adobe Photoshop (San Jose, CA).

### OCT Statistical Analysis

Linear regression mixed models were used to assess ONL and FRT in five groups representing each transgenic line and WT mice. Linear regression mixed models were used to account for the within-animal correlation that occurs when multiple observations are taken from the same subject. First, we examined whether the mean ONL and FRT across the entire retina differed within each transgenic line group over time (three weeks vs. ten weeks). T-tests were also used to examine this association at each eccentricity (±0.56 mm, ±0.42 mm, ±0.28 mm, ±0.14 mm). Next, we examined whether there were any between-group differences at each time point. Finally, a statistical interaction term was added to the model to assess if the changes over time differed by transgenic group. Separate linear mixed models were used to assess mean ONL and mean FRT. For five of the animals, data was collected from the same animal at both time points. Therefore, two separate analyses were employed to account for the paired nature of these measurements. First, data collected at ten weeks was treated as independent observations from those collected at time three. Second, the analyses were re-run excluding data collected at ten weeks for just those five animals. In both approaches, the pattern of results was similar. The results from the first analysis are presented; however, differences in results are noted where applicable. Adjustment of p-values for multiple comparisons was not performed. P-values < 0.05 were considered statistically significant.

### Histology

Morphologic analysis was performed as previously described^14^. Briefly, eyes were oriented, enucleated, and fixed in 2% paraformaldehyde containing 2.5% glutaraldehyde in 0.1 M PBS (pH 7.4). A slit was made in the cornea with a single edge blade and the lens/anterior chamber was removed and discarded. The eye cup was placed in the same fixative for two hours, followed by dehydration via graded alcohol concentrations. The eye cup was then moved to propylene oxide until it was embedded in an epoxy mixture of Embed 812 (Electron Microscopy Sciences). Semithin (0.5–1 μm) sections were cut with an ultramicrotome beginning nasally and cutting towards the temporal region. Sections used in representative images were from a region within 150 μm of the optic nerve. Sections were stained with 2% toluidine blue and light micrographs were obtained on a Zeiss Axioplan 2 microscope equipped with a Axiocam digital camera and Axiovision 4.6.3 software (Zeiss). Similarly to OCT measurements, ONL and FRT were measured for each genotype (n = 3) at 3 and 10 weeks at 8 eccentricities (±0.56, ±0.42, ±0.28, and ±0.14 mm) in fixed toluidine blue stained tissues. Measurements were performed in image J (NIH, Bethesda, MD)^50^. Ultrathin sections (80–90 nm) for electron microscopy (EM) were prepared as previously described^14^ and visualized on a JEOL 1200 electron microscope equipped with a digital camera (AMT Gatan, Inc; Pleasanton, CA; four megapixel; ES1000-785). Immunohistochemistry was performed as previously described using a custom designed purified antibody (Genscript) with the epitope (N-*Cngb1;* QEPPEPKDPPKPPGC) (1:50)^14^. The only adjustment made to the previously published protocol was primary and secondary antibody incubation at 37 °C and shortening of primary antibody incubation time from overnight to 6 ½ hours. The secondary antibody used was Donkey anti-rabbit Alexa-647 (Thermo Fisher Scientific, Waltham, MD) (1:500). Mice were between 4 and 6 weeks of age. All images were obtained on a Nikon A1 plus (Melville, NY) confocal at a constant laser intensity for each chromophore; brightness/contrast was adjusted identically for all images in Adobe Photoshop. Negative controls for each genotype were done exactly the same, but without primary antibody.

### Western Analysis

Western analysis on blots for GARP1 were performed as previously described^14^ using a custom designed affinity purified antibody (Genscript) with the epitope CVSRITPLPATSGTQYHG. Westerns were completed in the Wes automated Protein Simple system in order to determine expression levels of CNGB1 proteins. Retinas were dissected into homogenization buffer (10 mM Tris, pH 7.5, 0.5% Triton-X 100) and 1X Sigma Protease inhibitor cocktail (Roche, Florence, South Carolina) for all genotypes. A protein assay (BioRad) was performed to determine protein concentration and 0.15 mg/ml was used. CNGB1 protein expression was determined using an automated “Wes” Western blotting system (ProteinSimple, Inc., San Jose, CA, USA), The Wes is a capillary electrophoresis based immunodetection system that provides higher reproducibility at lower sample concentration, increased sensitivity, and higher resolution than traditional western blot protocols^51^. The Wes ProteinSimple system was used according to the manufacturer’s instructions, default settings were used. The N-terminal antibody (N-*Cngb1;* QEPPEPKDPPKPPGC) was used (1:500) to detect expression of the β-subunit and GARP2. N-*Cngb1* was generated in rabbit and affinity purified (Genscript Inc; Piscataway, NJ). Data analysis was performed using the Compass Software (ProteinSimple) and quantitation was determined by normalizing the area under the curve of the β-subunit and GARP2 peaks by the area under the curve of the ProteinSimple internal standard peak. Representative computer generated electerophoretic images were generated by the Compass Software. Images shown contain information from capillaries run over multiple trials. The computer generated electrophoretic images displayed larger product size than is normally observed for these proteins. The very low pI of the proteins is likely the basis for the altered migration, which is even more pronounced in the capillary than is observed in an acrylamide gel^51^. Previous reports comparing capillary based protein migration to standard SDS gels have also shown differences (either higher or lower) in size of proteins between the two techniques^52^.

### Electroretinography

Scotopic ERG responses were measured at 6 different intensities of light in each genotype at 3 weeks and 10 weeks of age. Mice to be used for full-field ERG analysis were dark adapted overnight, sedated with 3% isoflurane in a closed chamber, and anesthesia was maintained at 2% via a certified isoflurane vaporizer (EZ Anesthesia Euthanex Corp; Palmer, PA). Eyes were anesthetized with proparacaine drops (AKORN; Lake Forest, IL; 0.5%) and pupils dilated with topical phenylephrine HCL and tropicamide (AKORN; Lake Forest, IL; 1%). Only eyes receiving light stimuli were dilated. Methylcellulose 2.5% (Goniosol, CIBA Vision Corp, Duluth, GA) was applied to the corneal surface as well as to the contact lens placed on top of the silver-embedded recording electrode. The reference and ground electrodes were stainless steel subdermal needles placed in the cheek and lower back, respectively. During recordings the equipment and animal were enclosed in a Faraday cage and body temperature maintained at 37 °C by a digital heating pad placed under the animal. Defined light pulses between 0.1 cd*s/m^2^ and 25 cd*s/m^2^ were delivered through a Ganzfeld dome on an Ocuscience ERG (HMsERG Lab System; Ocuscience; Henderson, North Virginia) instrument manufactured for functional analysis of animal eyes. ERG data, including time/response and waveforms, were viewed using proprietary ERGView 4.860 A software and analyzed after extraction into Microsoft Excel 2013 (ver. 15.0.4763.1003). Standard t-tests were used to analyze differences in the maximum saturating a- and b-wave response (μV) between genotypes at each light intensity.

## Additional Information

**How to cite this article:** DeRamus, M. L. *et al*. GARP2 accelerates retinal degeneration in rod cGMP-gated cation channel β-subunit knockout mice. *Sci. Rep.* **7**, 42545; doi: 10.1038/srep42545 (2017).

**Publisher's note:** Springer Nature remains neutral with regard to jurisdictional claims in published maps and institutional affiliations.

## Supplementary Material

Supplementary Material
