# Supplementary material for: GARP2 accelerates retinal degeneration in rod cGMP-gated cation channel β-subunit knockout mice
Source: Sci Rep. 2017 Feb 15;7:42545. doi: 10.1038/srep42545 (PMC5309851; doi:10.1038/srep42545)
Supplement: Supplementary Material [file srep42545-s1.pdf]

GARP2 accelerates retinal degeneration in rod cGMP-gated cation channel β-subunit knockout mice

Marci L. DeRamus[1], Delores A. Stacks[1], Youwen Zhang[1], Carrie E. Huisingh[2], Gerald McGwin[3], *Steven J. Pittler[1]

Departments of [1]Optometry and Vision Science, School of Optometry, [2]Ophthalmology, School of Medicine, and [3]Epidemiology, School of Public Health, University of Alabama at Birmingham, Birmingham, AL 35294

Corresponding Author: Steven J. Pittler, 1670 University Blvd., Birmingham, AL 35523; phone 205-934-6744; e-mail pittler@uab.edu

Supplementary Material

Supplementary Table 1 Proteins expressed in the different genotypes

Genotype	β-subunit	GARP1	GARP2
WT	P	P, ND	P
$X1^{-/-}$	NP	NP	NP
$X26^{-/-}$	NP	P, ND	P, 18% of WT GARP2
$X1^{-/-}$ $G1^{Tg}$	NP	P, 185% of WT GARP2	NP
$X1^{-/-}$ $G2^{Tg}$	NP	NP	P, 42% of WT GARP2
$X1^{-/-}$ $G1^{Tg}$ $G2^{Tg}$	NP	P, 215% of WT GARP2	P, 204% of WT GARP2

P=present, NP=not present, ND=not detectable with N-terminal antibody

SupplementaryTable 2. Scotopic ERG responses in 3-week old mice

Intensity ($cd*s/m^2$)	0.0001	0.0003	1.00	3.00	10.00	25.00
			**a-wave (μV±SEM)**			
WT (5)	62 ± 18	115 ± 27	184 ± 32	241 ± 33	304 ± 31	358 ± 32
$X1^{-/-}$ (5)	4 ± 1*	7 ± 2*	15 ± 3*	11 ± 2*	24 ± 5*	34 ± 3*
$X26^{-/-}$ (3)	3 ± 2*	4 ± 2*	8 ± 3* †	11 ± 5*	10 ± 3* †	21 ± 12*
$X1^{-/-}$ $G1^{Tg}$ (4)	4 ± 2*	3 ± 2*	4 ± 1* †	2 ± 1* †	7 ± 3* †	27 ± 8*
$X1^{-/-}$ $G2^{Tg}$ (3)	5 ± 2*	5 ± 2*	5 ± 3* †	13 ± 5*	14 ± 10*	26 ± 9*
$X1^{-/-}$ $G1^{Tg}$ $G2^{Tg}$ (4)	4 ± 2*	13 ± 5*	14 ± 3*	13 ± 5*	33 ± 15*	40 ± 15*
			**b-wave (μV±SEM)**			
WT (5)	455 ± 93	597 ± 72	682 ± 60	753 ± 52	875 ± 53	953 ± 55
$X1^{-/-}$ (5)	86 ± 18*	146 ± 23*	223 ± 30*	276 ± 34*	358 ± 39*	377 ± 43*
$X26^{-/-}$ (3)	40 ± 3* †	72 ± 17* †	87 ± 18* †	131 ± 24* †	173 ± 36* †	163 ± 21* †
$X1^{-/-}$ $G1^{Tg}$ (4)	36 ± 5* †	63 ± 5* †	106 ± 8* †	160 ± 19* †	229 ± 29* †	282 ± 48*
$X1^{-/-}$ $G2^{Tg}$ (3)	59 ± 33* †	106 ± 57* †	148 ± 95* †	195 ± 115* †	260 ± 134* †	261 ± 128* †
$X1^{-/-}$ $G1^{Tg}$ $G2^{Tg}$ (4)	42 ± 14* †	92 ± 22*	127 ± 41* †	168 ± 55*	238 ± 92*	254 ± 91*

* $p < 0.001$ vs WT, † $p < 0.05$ vs $X1^{-/-}$

Supplementary Table 3. Scotopic ERG responses in 10-week old mice

Intensity (cd*s/m^2)	0.0001	0.0003	1.00	3.00	10.00	25.00
**a-wave (µV±SEM)**						
WT (7)	96 ± 17	160 ± 21	206 ± 24	244 ± 25	294 ± 26	358 ± 39
X1$^{-/-}$ (9)	10 ± 2*	11 ± 2*	15 ± 2*	19 ± 4*	25 ± 3*	32 ± 5*
X26$^{-/-}$ (3)	4 ± 1* †	9 ± 3*	7 ± 1* †	12 ± 1* †	15 ± 2* †	24 ± 2*
X1$^{-/-}$ G1^{Tg} (8)	6 ± 2* †	7 ± 1* †	8 ± 2* †	11 ± 2* †	11 ± 2* †	14 ± 3* †
X1$^{-/-}$ G2^{Tg} (7)	10 ± 3*	7 ± 1* †	9 ± 1* †	14 ± 4*	14 ± 2* †	18 ± 2* †
X1$^{-/-}$ G1^{Tg} G2^{Tg} (5)	9 ± 2*	8 ± 1*	9 ± 1* †	14 ± 2*	12 ± 3* †	19 ± 3* †
**b-wave (µV±SEM)**						
WT (7)	546 ± 81	637 ± 74	674 ± 72	697 ± 67	824 ± 61	878 ± 65
X1$^{-/-}$ (9)	76 ± 15*	111 ± 22*	139 ± 26*	144 ± 31*	182 ± 44*	202 ± 46*
X26$^{-/-}$ (3)	19 ± 5* †	55 ± 33*	75 ± 36*	102 ± 27*	123 ± 23*	141 ± 27*
X1$^{-/-}$ G1^{Tg} (8)	41 ± 10* †	54 ± 14* †	76 ± 18* †	94 ± 21*	120 ± 23*	133 ± 24*
X1$^{-/-}$ G2^{Tg} (7)	27 ± 7* †	42 ± 8* †	66 ± 12* †	82 ± 9* †	90 ± 13* †	105 ± 14* †
X1$^{-/-}$ G1^{Tg} G2^{Tg} (5)	27 ± 6* †	30 ± 4* †	49 ± 12* †	70 ± 13* †	107 ± 19* †	114 ± 12* †

* $p < 0.001$ vs WT, † $p < 0.05$ vs X1$^{-/-}$

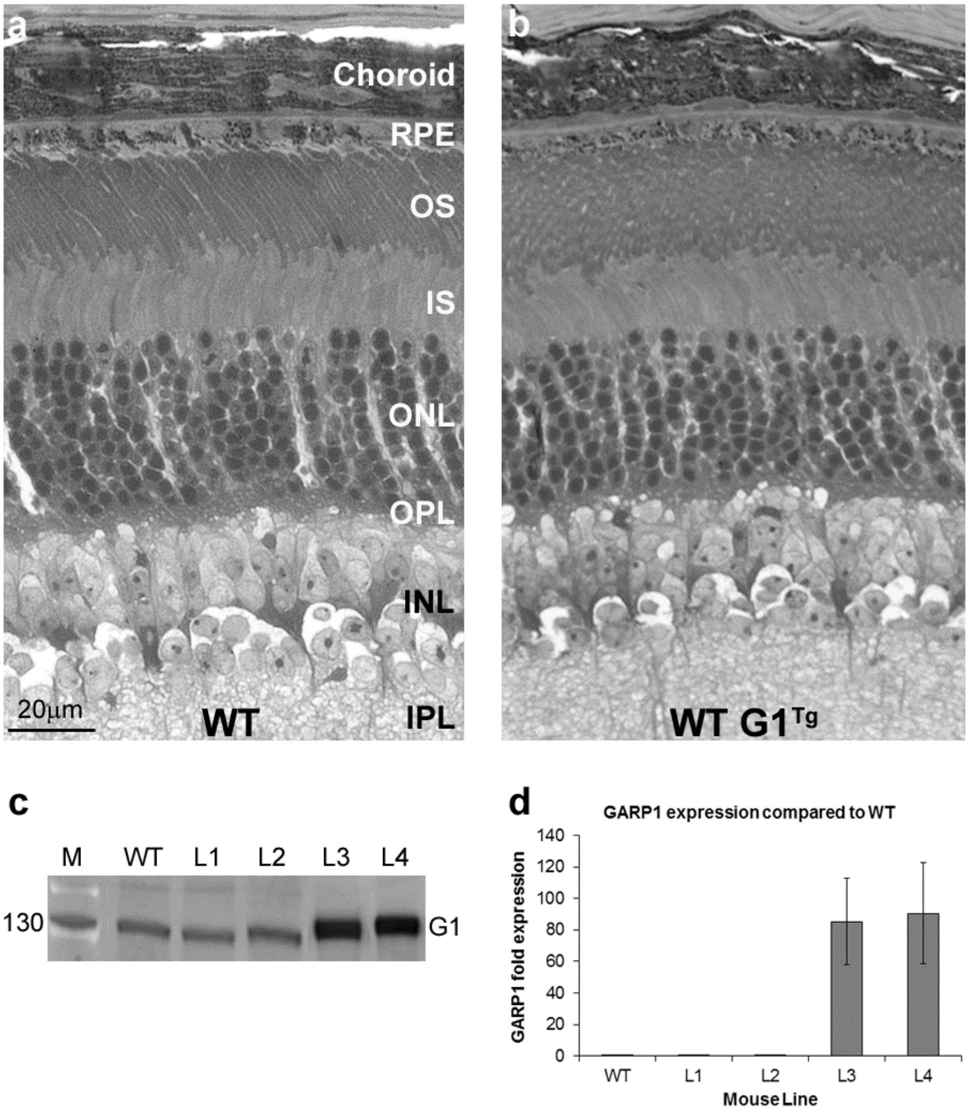

Supplementary Figure 1. Representative light microscopy images of 6 month old (a) WT and (b) GARP1 transgenic mice on a WT background aligned at the approximate region of the ELM. GARP1 transgenic mice maintain normal retina layer stratification and overall appearance. Full length GARP1 was inserted into a rhodopsin promoter construct and integrated into the mouse genome. (C) Western blot showing the four lines of mice identified. For quantitation 0.06 (WT, L1, L2) to 0.55 (L3 and L4) whole retina equivalents were loaded on 10% acrylamide gels. A 130kD band was observed

for GARP1 protein. (D) Lines 1 and line 2 on a WT background express GARP1 at levels similar to WT suggesting that the transgene may not be expressing much GARP1 in these lines. However, line 3 and line 4 express GARP1 85 and 91 fold over WT background levels, respectively. Based on these expression levels, L4 was used in the current study. RPE= retina pigment epithelium, OS= outer segment, IS= inner segment, ONL= outer nuclear layer, OPL= outer plexiform layer, INL= inner nuclear layer, IPL= inner plexiform layer. Scale bar = 20μm.

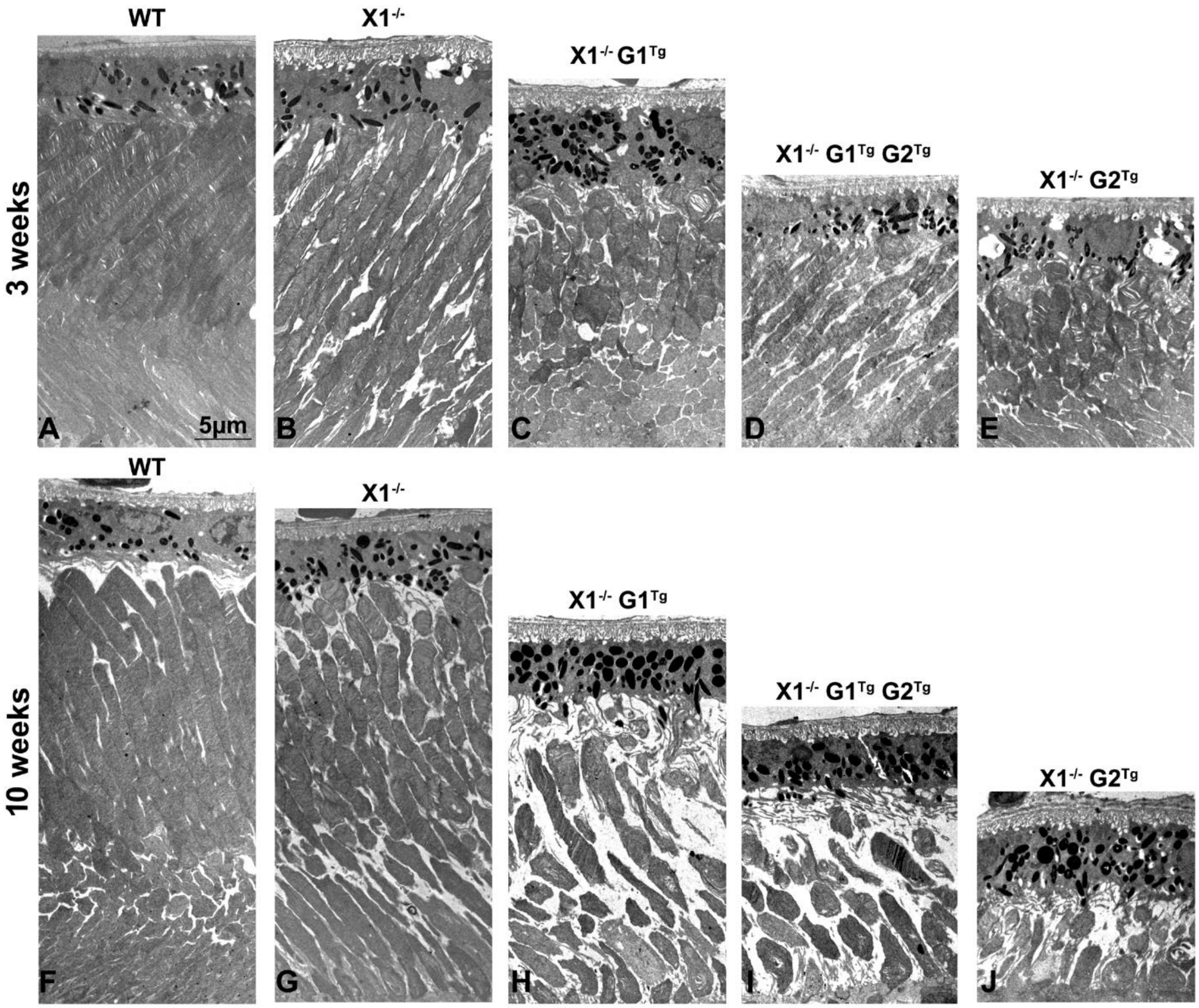

Supplementary Figure 2. Representative high magnification images of WT (A,F), $X1^{-/-}$ (B,G), $X1^{-/-}$ $G1^{Tg}$ (C,H), $X1^{-/-}$ $G1^{Tg}$ $G2^{Tg}$ (D,I), and $X1^{-/-}$ $G2^{Tg}$ (E,J) at 3 weeks (A-E) and 10 weeks (F-I). Scale bar = 5 µm. Consistent with the light microscopy, EM analysis shows increasing severity of retinal degeneration when GARPs are present (cf. G with H-J) and the greatest level of degeneration when only GARP2 is present (J).
